# Development of the Simplified Chinese version of neonatal palliative care attitude scale

**DOI:** 10.3389/fped.2022.962420

**Published:** 2022-09-27

**Authors:** Yajing Zhong, Beth Perry Black, Victoria J. Kain, Xiaoming Sun, Yang Song

**Affiliations:** ^1^Department of Public Health and Primary Care, Centre for Biomedical Ethics and Law, KU Leuven, Leuven, Belgium; ^2^School of Nursing, University of North Carolina at Chapel Hill, Chapel Hill, NC, United States; ^3^School of Nursing and Midwifery, Griffith University, Griffith, QLD, Australia; ^4^School of Nursing, Guangzhou University of Chinese Medicine, Guangzhou, China

**Keywords:** neonatal palliative care, neonatal nurses, Simplified Chinese, attitudes, scale development

## Abstract

**Background:**

The provision of palliative care for neonates who are not expected to survive has been slow in mainland China, and this model of care remains in its early stages. Evaluating nurses' attitudes toward neonatal palliative care (NPC) has the potential to provide valuable insight into barriers impeding NPC implementation. This study aimed to translate and adapt the traditional Chinese version of the Neonatal Palliative Care Attitude Scale (NiPCAS) into Simplified Chinese to assess its psychometric properties.

**Methods:**

The NiPCAS is a valid and reliable instrument to measure nurses' attitudes for evidence-based practice. To date, the scale has not been used largely in mainland China. With translation and cultural adaptation, the traditional Chinese version of the NiPCAS was developed into a Simplified Chinese version. Its reliability was tested using internal consistency and test-retest reliability, and its validity was measured using the content validity index and exploratory factor analysis.

**Results:**

A total of 595 neonatal nurses from mainland China were recruited. Twenty-six items in the scale were translated into Simplified Chinese. The scale demonstrated excellent reliability with a Cronbach's α coefficient of 0.87 and a test-retest reliability of 0.88. To support the Simplified Chinese version of NiPCAS, the scale content validity score was 0.98, and the exploratory factor analysis revealed five factors representing the conceptual dimensions of the scale.

**Conclusion:**

This study demonstrated the psychometric properties of the Simplified Chinese version of NiPCAS, validated its use as a viable tool for measuring neonatal nurses' attitudes toward NPC, and identified facilitators and barriers to NPC adoption. Our findings suggested supported clinical application in the context of mainland China. A confirmatory factor-analysis approach with a different sample of neonatal nurses is required for further testing of the instrument in the future.

## Introduction

Children are at the greatest risk of dying in the first 28 days of life, with 2.4 million children dying within the first month of life (1). The vast majority of neonatal deaths occur in low- and middle-income countries (1, 2). The United Nations Inter-agency Group for Child Mortality Estimation indicated that the neonatal mortality rate in China was 3.46%, while in Australia was 2.37% and in Japan was 0.85% (3). Such a high mortality rate suggested that healthcare professionals should raise their concern about newborns' quality of life and the impact upon families (4, 5). While technological advancement has improved survival rates, the number of children with complex chronic illnesses and disabilities continues to rise (6–9). Neonatal palliative care (NPC) improves the quality of life of dying babies (10), and there is a great need for NPC for newborns in China.

Every year, an estimated 21 million children, including newborns, may benefit from a palliative care approach (2, 11). NPC was defined as an active and holistic approach to alleviate suffering in neonates and the families facing problems associated with life-threatening conditions, which incorporated a multidisciplinary team and collaborative decision-making with the family (12, 13). Palliative care for neonates who are not expected to survive has been slow in mainland China, and this form of care is still in its infancy (14, 15). Little research has been conducted on NPC in mainland China, and healthcare professionals have little understanding about it (16–20).

Nurses play a vital role in NPC management given their responsibilities of advocating for the best interests of the neonates with life-limiting conditions as well as their families. Hence, understanding nurses' attitudes about NPC is key to the implementation of NPC practice in neonatal intensive care unit (NICU). However, in mainland China, very few researchers pose questions or conduct research in this topic, nurses' attitudes toward NPC are still unclear and to be discussed.

Kain (21) developed the Neonatal Palliative Care Attitude Scale (NiPCAS) to measure nurses' attitudes toward NPC. Psychometric validation of the NiPCAS has also been proven satisfactory in further studies in countries and regions including Australia (21), United States (22–24), Italy (25), Southeast Iran (26), Czech Republic (27), Turkey (28, 29), Portugal (30), and Taiwan (31). In order to explore the attitudes of NICU clinicians in mainland China, Gu and colleagues translated the traditional Chinese version of the NiPCAS into Simplified Chinese (32). However, Gu et al. (32) did not further validate or adjust the scale.

Simplified Chinese is the standard written form of Chinese used in mainland China, while traditional Chinese used more often in Taiwan, Hong Kong and Macao. Many nurses working in mainland China can only read or write Simplified Chinese characters. Additionally, some expressions of these two Chinese characters are different, even though they come from the same language. Hence, a validated Simplified Chinese scale is needed to further measure NICU nurses' attitudes toward NPC. Therefore, we aim to translate and adapt the *traditional* Chinese version of NiPCAS into a *Simplified* Chinese version, and to use this new scale for measuring nurses' attitudes toward NPC.

## Methods

### Design

This study consisted of two phases: translation (translation and cultural adaptation) and validation. We translated the traditional Chinese version of NiPCAS into a preliminary Simplified Chinese version, and adapted it to fit the expression habits in mainland China. We then administered it to neonatal nurses for twice as the pilot testing. Participants completed the second round of the questionnaires (T2) 4 weeks after the first (T1). Because of the 4-week interval between T1 and T2, individuals could forget their answers from the first round (T1) while maintaining the same attitude. We also interviewed five nurses to determine the readability of the questionnaire. Then we examined reliability and validity of the Simplified Chinese version of NiPCAS. Questionnaires were administered in hospital NICUs or through WeChat (an app for communication used in mainland China).

### Sample

We recruited neonatal nurses who worked in NICUs via convenience sampling. We expected to reach a sample size of 200 participants as was recommended to conduct an exploratory factor analysis (EFA) (33). However, in this study, an adequate sample size was vital in determining the properties of the translated instrument. In addition, the accuracy of the psychometric properties was reliant on sample size (34–36). Therefore, neonatal nurses were recruited *regardless* of their work experience to obtain a large a sample size as possible.

### Instrument

The original NiPCAS instrument included eight demographic questions and 26 attitude questions using a Likert scale. These items were categorized into three subscales: “Organization,” “Resources” and “Clinicians,” each of which had acceptable Cronbach α scores: 0.73, 0.65, and 0.63, respectively. Chen translated the 26 attitude items of the NiPCAS into traditional Chinese and demonstrated high content validity of CVI = 0.90 (31). The traditional Chinese version of scale was divided into four subscales: “Organization,” “Resources” “Clinicians,” and “Special Work Experiences and Beliefs.” We would develop the Simplified Chinese version of the NiPCAS.

### Reliability and validity

Cronbach's α coefficient and test-retest method were used to evaluate the scale reliability. Content validity and construct validity were measured to determine the items' congruence with the instrument.

### Data analysis

Statistical analyses were performed using SPSS version 24.0 (SPSS Inc., Chicago, IL). The demographic characteristics of the neonatal nurses were summarized with descriptive statistics.

Internal consistency reliability of the preliminary Simplified Chinese NiPCAS and its subscales were tested using Cronbach's α coefficient. Pearson's r was used to evaluate test-retest reliability. In the aspect of Cronbach's α coefficient, it would be considered acceptable if α value of the total scale was >0.70. Pearson's r scores for test and retest reliability were considered valuable if *r* >0.7 (37, 38).

Content validity of the scale was examined through a content validity index (CVI) based on expert consultation. An expert panel was assembled for evaluation of content validity. Experts made comments on the relevance and clarity of each item of the tool using 4-point Likert scale correspondingly: 1 = not relevant, 2 = somewhat relevant, 3 = relevant, 4 = highly relevant. The CVI was calculated at item level (item content validity index, I-CVI) and scale level (scale content validity index, S-CVI), with index values over 0.80 as recommended (39).

To establish the structure of the scale for construct validity, EFA was used. Factor extraction was performed using a principal component analysis approach (PCA) with direct oblimin rotation, in order to find out the uncorrelated principal components and to reach dimensionality reduction. Factors were extracted where eigenvalues were >1.0, and factor loadings above 0.40 were retained (40).

## Results

### Descriptive statistics

In the study, we administered the scale to 595 neonatal nurses from 40 NICUs in five provinces of China (Guangdong, Guizhou, Hebei, Henan, Hunan) and 92.5% (*n* = 550) completed the survey. Among the 550 nurses, 98.5% (*n* = 542) were female; ages ranged between 20 and 49 years old (M = 29); years working in NICUs ranged from 1 to 28 years (M = 6). 68.5% (*n* = 377) of nurses reported previous experience in caring for dying newborns; 56.3% (*n* = 310) of nurses had a college degree or above; nurses with religious beliefs accounted for 31.5% (*n* = 173). Nurses working in tertiary hospitals accounted for 86% (*n* = 473), secondary hospitals 9.8% (*n* = 54), and primary, private or other hospitals 4.2% (*n* = 23). Table 1 summarizes the demographic characteristics of the participants (41).

**Table 1:** Personal characteristics (*N* = 550) (41).

**Variables**		**N (%)**
Gender	Male	8 (1.5)
	Female	542 (98.5)
Age (years)	20–29	308 (56)
	30–39	198 (36)
	40–49	44 (8)
Working years	1–5	306 (55.6)
	6–10	154 (28.0)
	≥10	90 (16.4)
Experience of caring for dying neonates	Yes	377 (68.5)
	No	173 (31.5)
Degree	Technical secondary	21 (3.8)
	Junior college	219 (39.8)
	Undergraduate	306 (55.6)
	Graduate	4 (0.7)
Religion	Yes	174 (31.3)
	No	376 (68.4)
Hospital level	III	473 (86.0)
	II	54 (9.8)
	Private or others	23 (4.2)

### Forward translation and adaption

We translated and adapted the traditional Chinese version of the NiPCAS into a Simplified Chinese version with the approval of the original author (Kain) and original translator (Peng). Eight items (3, 17, 20, 21, 22, 23, 25, and 26) were designed to identify barriers to NPC and the other items were designed to identify facilitators to NPC. The Likert 5-point response scale (ranging from ‘strong agreement' to ‘strong disagreement') were coded thus: strongly disagree = 1; somewhat disagree = 2; unsure = 3; somewhat agree = 4; and strongly agree = 5. The “unsure” response was placed on the far right to discourage participants from habitually selecting that option, as in the original instrument. Higher mean scores correlated to more ‘positive' attitudes toward NPC. In addition to translation, we added eight demographic questions to the survey (see Table 1).

### Pilot testing

For pilot testing, we recruited other 15 neonatal nurses who worked at tertiary hospitals to complete questionnaires and retested 4 weeks later. Cronbach's α was 0.87 in the first round of testing and 0.88 in the second round, while the Pearson's r value at re-test was 0.89. To gather additional feedback from the nurses about the instrument, we read items to five of the participants one at a time and asked if they understood. Participants reported that the items on the scale were clear and easy to understand, and that the preliminary scale's expression was consistent with people's language habits in mainland China.

### Internal consistency

The Cronbach's α value of the preliminary Simplified Chinese version of NiPCAS was 0.87, and the subscales: “Organization,” “Resources,” “Special Work and Experiences,” and “Clinicians” were 0.80, 0.83, 0.58, and 0.29, respectively. The scale's overall Cronbach's α value was satisfactory, however the Cronbach's α value for “Clinicians” was comparatively lower. This could be explained by the fact that the “Clinicians” subscale only has two items, and we assumed that its internal consistency was not accurate enough. The two items situated in “Clinicians” were “Staff go beyond what they feel comfortable with in using technological life support” and “Staff are asked by parents to continue life-extending care beyond what they feel is right,” and they were proved to be relevant to clincians' attitudes toward NPC (21–32). After careful discussion, all items were retained.

### Test-retest reliability

The overall test-retest cronbach's α value was 0.88, Pearson's r was 0.89 (*P* < 0.000). Test-retest Cronbach's α values for each subscale were: 0.88 (Organization), 0.56 (Resources), 0.79 (Special Work and Experiences), 0.52 (Clinicians). Test-retest reliability was deemed acceptable.

### Content validity

Eight experts, including two neonatal physicians, three neonatal nurses, three professors with expertise in instrument development, palliative care and psychology, were invited to form an expert panel and provide advice regarding translation and rated content relevance on each item based on the Likert 5-point response scale. The first round of S-CVI was 0.83, I-CVI ranged between 0.43 and 1.00. Five items (4, 17, 20, 23, 25) received low scores and these items demonstrated weak correlation to the scale. The translation and expression were then revised and the second round of S-CVI was 0.98, I-CVI ranging between 0.83 and 1.00, indicating excellent content validity. Supplementary File 1 displayed the full adaption.

### Construct validity

#### Item analysis

To measure the correlation between items and scale, we used 4 methods to conduct item analysis. (1) Critical ratio: critical ratio of all items was >3.5 (CR = 3.8–17.2, *P* < 0.000), which demonstrated that all items could be included in the EFA. (2) Corrected item total correlation (CITC): CITC has also been used to test the correlation of items and the scale (*r* = 0.28–0.65), items 3, 17, 20, 23, 25, 26 had lower CITC (*r* < 0.40). (3) Inconsistency: the inconsistency of the scale was high (Cronbach's α = 0.87, CITC = 0.21-.60), but the CITC of items 1, 2, 3, 11, 17, 20, 21, 23, and 26 were < 0.40. (4) Communities: communities of items 1, 3, 10, 11, 17, 21, 22, 23, 24, 25 and 26 were < 0.20. Factor loading ranged from 0.21 to 0.71; items 3, 10, 17, 20, 23, 25, and 26 were < 0.40. Results of these four tests were inconsistent. We discussed with the expert panel and agreed that the scale items were theoretically highly correlated. The overall scale was homogeneous, which was suitable for measuring neonatal nurses' attitude. The reliability and validity of the original and traditional Chinese version scale were acceptable, so we decided to retain all items to conduct the EFA.

#### Exploratory factor analysis (EFA)

**Figure 1:**
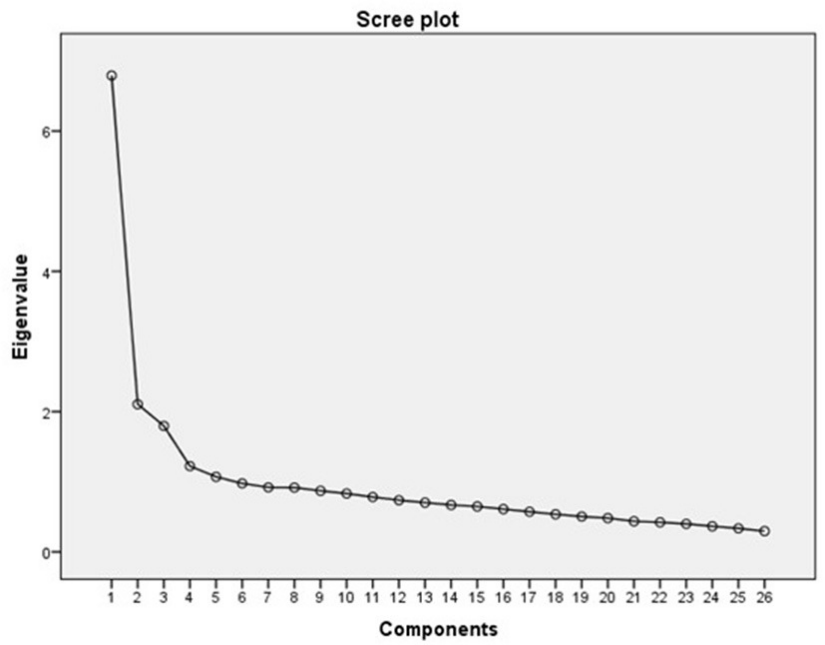
Scree plot.

The NiPCAS items were factor analyzed using the principal components method of factor extraction. Kaiser–Meyer–Olkin statistic was 0.89, and Bartlett's test of sphericity was statistically significant (χ2 = 4185.79, *p* < 0.000). Using a minimum eigenvalue of 1.0 as the criterion for factors, five factors, accounting for 49.95% of the variance, were extracted. The scree plot demonstrated that the scale teetered downwards after the fourth factor (Figure 1), so we extracted four factors. PCA was performed with direct oblimin rotation to identify derivative factors given the variable correlation between items and scale. The percentage of variance explained was 45.83%, and items with factor loadings ≥ 0.40 were retained. If an item loaded onto different factors, theoretical interpretation and parsimony were considered. Factor loading of item 3 and item 26 were lower than 0.40, however they were retained after discussion with the expert panel since they were considered useful in identifying barriers to NPC implementation. The rotated solution demonstrated the presence of simple structure, with most components demonstrated several strong loadings and a tendency to load uniquely onto only one factor. The items in Table 2 were ordered and blocked by size of loading to facilitate interpretation of the factor matrix.

**Table 2:** Rotated factor matrix for PCA of the translated NiPCAS.

**Items**	**Factor 1**	**Factor 2**	**Factor 3**	**Factor 4**
14	0.786	0.169	0.050	0.205
7	0.716	0.170	0.243	0.397
13	0.701	0.308	0.055	0.203
6	0.688	0.155	0.303	0.445
15	0.686	0.214	391	0.130
16	0.661	0.187	0.419	0.032
18	0.657	0.350	0.031	0.279
9	0.613	0.219	0.245	0.573
24	0.600	0.201	0.059	−0.052
19	0.583	0.251	0.490	0.240
8	0.512	0.081	0.460	0.169
21	0.131	0.640	0.323	0.043
25	0.213	0.585	−0.043	0.135
20	0.153	0.584	0.126	0.091
23	0.209	0.569	−0.351	0.030
17	0.068	0.552	0.222	−0.167
22	0.342	0.527	0.200	−0.047
11	0.242	0.423	0.111	0.211
26	0.235	0.392	0.011	−0.247
12	0.245	0.170	0.742	0.141
1	0.230	0.113	0.590	0.299
10	0.255	0.324	0.561	0.077
3	0.137	0.363	0.373	−0.285
2	0.300	0.186	0.161	0.705
5	0.425	0.177	0.517	0.565
4	0.312	0.263	0.398	0.525

The scale had been separated into four subscales, “Organization and Resources,” “Barriers,” “Work Experience” and “Beliefs.” Based on the literature (20), the subscale “Organization and Resources” was further divided into separate subscales “Organization” and “Resources,” resulting in a five-factor solution. Subscale “Organization” included item 5, item 8, item 15, item 16, item 19. Subscale “Resources” comprised item 6, item 7, item 13, item 14 and item 24. Subscale “Barriers” was composed of item 3, item 17, item 20, item 22, item 23, item 25, and item 26. Subscale “Work Experience” contained item 2, item 11 and item 18. Other items including item 1, item 4, item 10, and item 12 belonged to subscale “Beliefs.”

## Discussion

We translated and cross-culturally adapted the NiPCAS into a Simplified Chinese version following disciplined approaches and examined its validity and reliability with an adequate sample size of neonatal nurses in mainland China.

In terms of reliability, the scale has good internal consistency. The high Cronbach's α value and Pearson's r value for the total scale also demonstrated its high stability over time. The Cronbach's α value for the ‘Clinicians' subscale was lower, but we assumed that this was because the subscale contained only two items. Regarding test-retest reliability, the overall reliability was adequate. Therefore, this new version of NiPCAS is deemed to be reliable. Our findings were consistent with other studies that got acceptable reliability of Cronbach α ranging from 0.43 to 0.87 (21–29, 31, 32). In Sousa et al. (30), the Cronbach α of the subscale “Clinicians” was.30, which was unacceptable. However, authors still kept this subscale because of the same reason that the subscale just contained two items.

In this study, we examined content validity and modified translations based on the expert panel's suggestions. The scale had excellent content validity and was recommended to be applicable to clinical nurses. The results were also consistent with other conducted studies that with CVI of 0.85 to 0.90 (26, 31, 32), or content validity ratio of 0.42 to 1.00 (27, 28).

The EFA yielded an interpretable five-factor solution, which accounted for 45.83% of variance. The five-factor structure found in this study was inconsistent with the former traditional Chinese version of NiPCAS. Items in the “Organization” and “Resources” subscales were the same as the traditional Chinese version. However, we built three different subscales, including “Barriers,” “Work Experience,” and “Beliefs.” These five factors together constituted the Simplified Chinese version of NiPCAS with an acceptable validity.

In addition, an English version, the Iranian version and the Czech version of NiPCAS created other subscales. In Chin et al. (24), subscales of Unite Culture, Resources, and Perceived Inappropriate Care were found after performing EFA. In Forouzi et al. (26), subscales of Insufficient Resources, Inappropriate Personal and Social Attitudes, Inappropriate Organizational Culture, and Inadequate Nursing Proficiency were formed after measuring content validity. Moreover, in Kachlová et al. (27), Organization, Resources, Doctors, and Experience and Attitudes of Nurses subscales were created after expert consult.

Dimensions of our adapted version of the scale were different from the traditional Chinese version or other language versions (21–32). Homogeneity of some items was also controversial but none items were suggested for exclusion by the expert panel; therefore, we still retained all items. However, it was almost impossible for a measurement to have perfect reliability and validity (42, 43). Psychometric properties for the measurement were only a general rule. Therefore, we focused more on overall results of the scale and items, rather than making comparisons with other versions.

Overall, our study proved the reliability and the validity of the Simplified Chinese version of the NiPCAS which could be used for measuring neonatal nurses' attitudes toward NPC. Applying the scale in the Chinese context is necessary to further verify its psychometric properties, understand nurses' attitudes and to develop the NPC practices in mainland China.

### Limitations

Although sample size in this study met the requirements for testing reliability and validity of the scale, representation of the sample and generalizability of the research results were limited given the questionnaire were administered in only five provinces. Some items had lower reliability or validity though the overall value of the scale was acceptable. Some items had lower factor loadings but were maintained via consensus. Despite these limitations, our study demonstrated that the Simplified Chinese version of NiPCAS was a reliable and valid instrument.

## Conclusion

This study evaluated the Simplified Chinese version of NiPCAS and demonstrated evidence of several valuable psychometric properties of the scale. Our analysis suggested that this tool has acceptable reliability and validity, which could be a concise and useful instrument for neonatal nurses to measure their attitudes toward NPC in mainland Chinese practice settings, and to understand influencing facilitators and barriers to NPC implementation. We have added evidence of the measurement properties and contributed to a wider application in international clinical nursing contexts. By applying this measurement in clinical practice and understanding nurses' perspectives about NPC, we would have a good starting point to improve NPC development in mainland China. A confirmatory factor-analysis approach should be conducted to further test this Simplified Chinese version of NiPCAS in the next step.

## Data availability statement

The original contributions presented in the study are included in the article/Supplementary material, further inquiries can be directed to the corresponding author/s.

## Ethics statement

The studies involving human participants were reviewed and exempted by First Affiliated Hospital of Guangzhou University of Chinese Medicine. We obtained informed consent from participants, assured participants of their anonymity, and informed them that they could withdraw from the study at any time without explanation. All data were processed to protect participants' anonymity.

## Author contributions

YZ designed and implemented the study, analyzed data, and drafted the manuscript. BB designed and supervised the study, commented, edited, and polished the manuscript. VK commented, edited, and polished the manuscript. XS edited and polished the manuscript. YS designed and supervised the study, commented, and edited the manuscript. All authors contributed to the article and approved the submitted version.

## Funding

The study was supported by a grant from Guangdong Academic of Social Science (Grant No. GD20CGL06) and Guangzhou University of Chinese Medicine (Grant No. 2022ZDPY01).

## Conflict of interest

The authors declare that the research was conducted in the absence of any commercial or financial relationships that could be construed as a potential conflict of interest.

## Publisher's note

All claims expressed in this article are solely those of the authors and do not necessarily represent those of their affiliated organizations, or those of the publisher, the editors and the reviewers. Any product that may be evaluated in this article, or claim that may be made by its manufacturer, is not guaranteed or endorsed by the publisher.
